# Bacterial pathogens and resistance causing community acquired paediatric bloodstream infections in low- and middle-income countries: a systematic review and meta-analysis

**DOI:** 10.1186/s13756-019-0673-5

**Published:** 2019-12-30

**Authors:** Nina Droz, Yingfen Hsia, Sally Ellis, Angela Dramowski, Mike Sharland, Romain Basmaci

**Affiliations:** 1 Service de Pédiatrie-Urgences, Hôpital Louis-Mourier, APHP, 178 rue des Renouillers, 92700 Colombes, France; 2 Paediatric Infectious Diseases Research Group, Institute of Infection and Immunity, St George’s, University of London, London, UK; 3 Global Antibiotic Research and Development Partnership, Geneva, Switzerland; 4 Department of Paediatrics and Child Health, Division of Paediatric Infectious Diseases, Faculty of Medicine and Health Sciences, Stellenbosch University, Cape Town, South Africa; 5 Université de Paris, INSERM, Infection, Antimicrobiens, Modélisation, Evolution (IAME), F-75018 Paris, France

**Keywords:** Bloodstream infection, Resource-limited settings, Children, Antimicrobial resistance, Epidemiology, Sepsis, Bacteraemia

## Abstract

**Background:**

Despite a high mortality rate in childhood, there is limited evidence on the causes and outcomes of paediatric bloodstream infections from low- and middle-income countries (LMICs). We conducted a systematic review and meta-analysis to characterize the bacterial causes of paediatric bloodstream infections in LMICs and their resistance profile.

**Methods:**

We searched Pubmed and Embase databases between January 1st 1990 and October 30th 2019, combining MeSH and free-text terms for “sepsis” and “low-middle-income countries” in children. Two reviewers screened articles and performed data extraction to identify studies investigating children (1 month-18 years), with at least one blood culture. The main outcomes of interests were the rate of positive blood cultures, the distribution of bacterial pathogens, the resistance patterns and the case-fatality rate. The proportions obtained from each study were pooled using the Freeman-Tukey double arcsine transformation, and a random-effect meta-analysis model was used.

**Results:**

We identified 2403 eligible studies, 17 were included in the final review including 52,915 children (11 in Africa and 6 in Asia). The overall percentage of positive blood culture was 19.1% [95% CI: 12.0–27.5%]; 15.5% [8.4–24.4%] in Africa and 28.0% [13.2–45.8%] in Asia. A total of 4836 bacterial isolates were included in the studies; 2974 were Gram-negative (63.9% [52.2–74.9]) and 1858 were Gram-positive (35.8% [24.9–47.5]). In Asia, *Salmonella* typhi (26.2%) was the most commonly isolated pathogen, followed by *Staphylococcus aureus* (7.7%) whereas in Africa, *S. aureus* (17.8%) and *Streptococcus pneumoniae* (16.8%) were predominant followed by *Escherichia coli* (10.7%). *S. aureus* was more likely resistant to methicillin in Africa (29.5% vs. 7.9%), whereas *E. coli* was more frequently resistant to third-generation cephalosporins (31.2% vs. 21.2%), amikacin (29.6% vs. 0%) and ciprofloxacin (36.7% vs. 0%) in Asia. The overall estimate for case-fatality rate among 8 studies was 12.7% [6.6–20.2%]. Underlying conditions, such as malnutrition or HIV infection were assessed as a factor associated with bacteraemia in 4 studies each.

**Conclusions:**

We observed a marked variation in pathogen distribution and their resistance profiles between Asia and Africa. Very limited data is available on underlying risk factors for bacteraemia, patterns of treatment of multidrug-resistant infections and predictors of adverse outcomes.

## Background

Estimated global childhood mortality has declined from 9.5 million to 7 million deaths annually over the past decade. This is largely due to the reduction of mortality in certain high-burden infectious diseases including diarrhea, pneumonia, malaria and measles [1]. However, sepsis remains the second leading cause of death with an estimated mortality rate of 7% in the paediatric population [2]. Most of these deaths were reported from facilities in Sub-Saharan Africa and Asia, where healthcare access, infrastructure and staffing remains sub-optimal. Bacterial infections, such as lower respiratory tract infection, meningitis and other infectious diseases, remain the leading causes of death in these regions [3]. Of note, epidemiology of bacterial infections is different across the world: studies of children with bacteraemia in Africa suggest that prevalence of bacterial infections among inpatients with bacteraemia is greater than that described in wealthier regions [4–6].

In resource-limited settings, the emerging threat of multidrug resistance among Gram-negative bacteria (GNB) is a major concern, given the scarcity of diagnostic microbiology laboratories and difficulty in accessing effective antibiotic therapy for resistant pathogens. Rising antibiotic resistance rates among *E. coli* (with resistance to third-generation cephalosporins and fluoroquinolones) is particularly problematic, since cephalosporins are the mainstay of empiric therapy for both community-acquired and hospital-acquired bloodstream infection in resource-limited settings.

The estimated prevalence of extended-spectrum beta-lactamase (ESBL)-producing *Enterobacteriaceae* in Asia and Sub-Saharan Africa is between 60 and 90% [7], highlighting the growing challenge of treating bloodstream infections in these countries. In May 2017, the United Nations World Health Assembly and World Health Organization (WHO) approved a resolution to tackle sepsis and made it a global health priority in the next decade [8].

The current WHO guideline recommends the combination of ampicillin and gentamicin for empiric treatment of paediatric sepsis. The second-line antibiotic recommended is a third-generation cephalosporin, or when staphylococcal infection is suspected, flucloxacillin and gentamicin should be considered [9]. Despite the above recommendations, many low- and middle-income countries (LMIC) utilize third-generation cephalosporins as first-line treatment for severe sepsis owing to their affordability and widespread availability [10].

Only very limited data on the etiology, epidemiology and antimicrobial susceptibility of the key pathogens are available regarding paediatric bacteraemia in low- and middle-income countries (LMIC) [11]. Given the paucity of epidemiological data on bacteraemia in children, we undertook a systematic review and meta-analysis, characterizing community-acquired paediatric bacteraemia in LMIC settings, including identification of key pathogens and antimicrobial resistance patterns.

## Methods

### Search strategy and selection criteria

Studies were considered eligible for inclusion if they reported children with community-acquired bloodstream infections, as defined by authors, which were laboratory-confirmed with a positive blood culture, from low and middle-income countries. Furthermore, studies had to include (i) infants or children aged > 1 month but less than 18 years of age; (ii) infants/children with submission of at least one aerobic blood culture; and (iii) data reporting the total number of pathogenic bacteria isolated. Countries were classified based on income using World Bank categories [12].

Pubmed and Embase databases were systematically searched for studies reported between January 1st 1990 and October 30th 2019. Pubmed was searched with a strategy combining MeSH (Medical Subject Headings) and free text: (sepsis OR bacterem* OR bacterae* OR septicaem* OR septicem* OR fever OR “bloodstream infection”) AND (“developing countri*” OR “under-developed nations” OR “third-world countr*” OR “third-world nation” OR “Resource-limited setting” OR “low-middle-income countr*” OR “low-income countr*” OR “middle-income countr*”). The detailed search strategy used in Embase is described in Additional file 1. Search strategy was restricted to English language. The study protocol was registered in PROSPERO (International prospective register of systematic reviews) under number 100367.

Two reviewers (ND, RB) performed the electronic searches and screened the titles and abstracts, independently. Studies that did not meet eligibility criteria were rejected on initial review. Articles marked for potential inclusion were obtained electronically or in paper copy and assessed again for inclusion. Any disagreements over the eligibility of particular studies were resolved through discussion with a third reviewer (MS).

We excluded studies that reported only hospital acquired infections, only assessed bloodstream infection in specific risk group (e.g. neutropenia, sickle cell anaemia), investigated a specific clinical syndrome (e.g. pneumonia, meningitis), or studies that were not from LMIC. We also excluded systematic reviews, case reports, editorials, policy statements, and studies during epidemic or outbreak. As we aimed to focus only on paediatric sepsis, we excluded studies where data on pathogen distribution and antibiotic resistance patterns were pooled i.e. not distinguishable between neonates, infants, older children, adolescents and adults. We excluded studies that focused on neonates since the epidemiology of neonatal infections is different to that of older children. Moreover, a review of community-acquired pathogens in neonatal sepsis in Asia and Africa has already been published [13].

Finally, we excluded studies that included only fungal infections or studies that included culture sites other than blood, where the results could not be separated by site of specimen.

### Quality assessment

To score the quality of eligible publications, we used the STrengthening the Reporting of OBservational studies in Epidemiology (STROBE) Statement on items that should be included in reports of cohort studies [14]. We calculated the proportion of items of the STROBE checklist adequately reported for each study. We did not exclude any studies based on quality.

### Data extraction

Descriptive and quantitative data from each included paper were extracted individually by two reviewers (ND and RB). Information for extractions included: hospital setting, country and region of the study, years the study was carried out, study period, inclusion criteria used in each study, blood cultures techniques, age range, number of patients who had blood cultures sampled, the number who had positive cultures for a bacterial pathogen, pathogens and contaminants isolated, and when available: mortality rate, co-infection with malaria at BSI diagnosis, prevalence of malnutrition, HIV status, and antimicrobial susceptibilities.

Data on blood culture contamination rate and most prevalent blood culture contaminants was not provided in almost all included papers and we were unable to include this factor in the final analysis.

### Statistical analysis

We performed a meta-analysis to estimate the rate of bacteraemia, the proportional representation of each pathogen and the case-fatality rate. A random-effect meta-analysis model was used to control for the inter-study variability effect. The proportions obtained from each study were pooled using the Freeman-Tukey double arcsine transformation and generated forest plots [15]. A *p* value < 0.05 was considered to be statistically significant. *I*^*2*^ statistic was used to determine heterogeneity [16]. Low, moderate, and high heterogeneity was defined to levels of *I*^*2*^ values of 25, 50, and 75% respectively [16]. We also performed a subgroup meta-analysis for each pathogen and by continent. All statistical tests were performed with R statistical package 3.3.2 (R Foundation for Statistical Computing, Vienne, Austria).

## Results

### Study selection and description

**Fig. 1:**
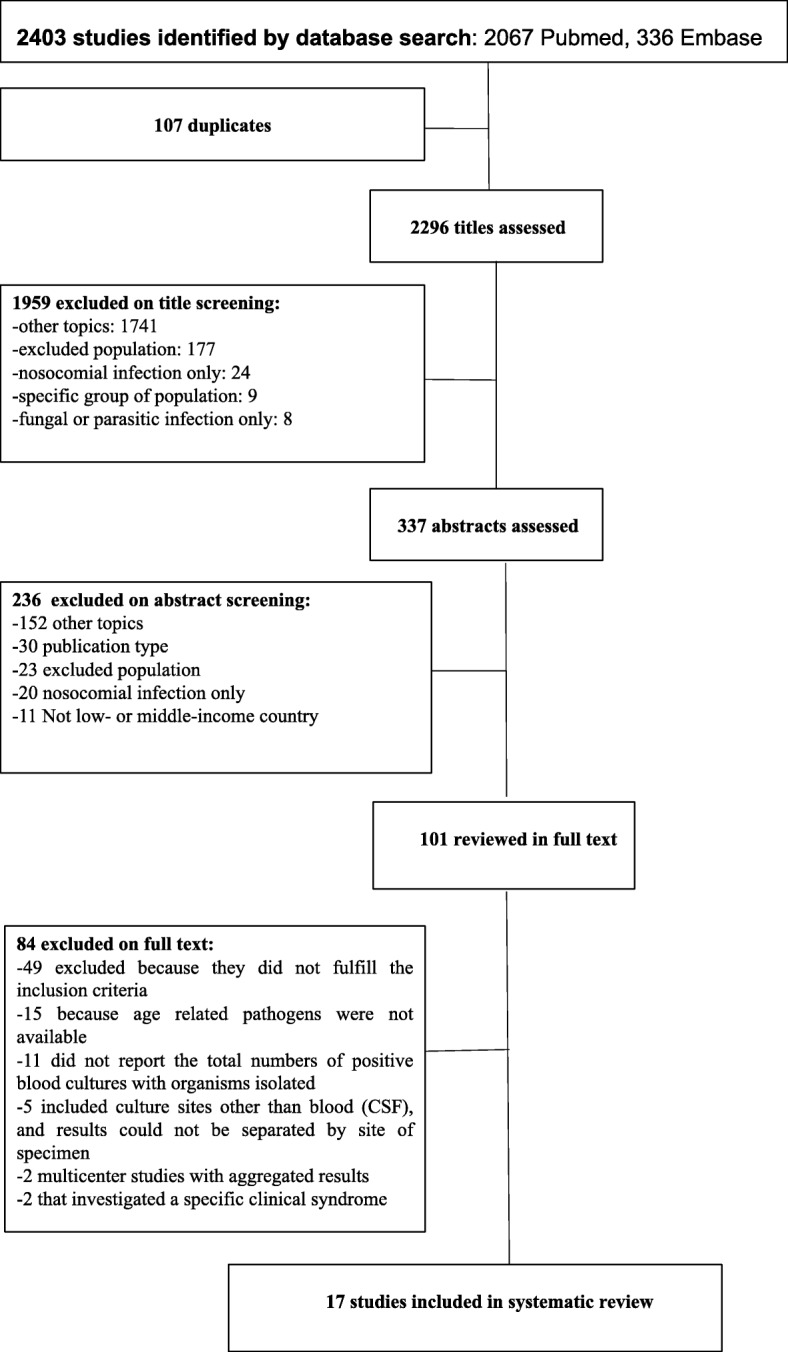
Diagram for study selection

We identified 2403 potentially relevant studies through the database search. Of these, there were 107 duplicates and 2195 were excluded on basis of title and abstract screening. A total of 17 studies [17–33] were included in the final review (Fig. 1); they were conducted in 12 countries (6 in Africa and 6 in Asia) between 1988 and 2011 and published between 1992 and 2016 (Additional file 1: Tables S1a and S1b). No studies from regions other than Africa and Asia, or any studies conducted after 2011 were eligible based on these inclusion criteria.

### Quality assessment

Overall, the published studies reported on a median of 66% (range 50–81%) of STROBE items (Additional file 1: Tables S1a and S1b).

### Study designs

All studies included were observational cohort studies, 16 were prospective [17–31, 33] and 1 was retrospective [32]. The median duration of the study period was 32 months (IQR 12–48).

Two studies [22, 25] investigated the etiology of positive blood cultures in all child admissions (11.8%), 11 [17–21, 23, 26–28, 30, 33] only in children admitted with fever without localizing features (64.7%) and 4 [24, 29, 31, 32] studies reported bloodstream infections in children admitted with signs of severe illness or suspected sepsis (23%).

Five studies [18, 23, 25, 31, 32] specifically defined community-acquired bacteraemia as clinically relevant positive blood cultures taken within 48 h of hospital admission or if blood culture was taken after 48 h and the clinical presentation and identified pathogen were consistent with community-acquired disease. Eleven other studies [13, 15–18, 20, 22, 24–26, 29] reported community-acquired bacteraemia exclusively, but did not give a clear definition of community-acquired bloodstream infection. One study [27] reported both community-acquired (90%) and hospital-acquired (10%) infections.

Ten studies (58.8%) [18–20, 22, 25, 26, 28–31] reported the volume of blood specimens sampled from patients. The culture media and methods of identification of organisms varied between studies with minimum culture volumes ranging from 1 mL to 5 mL. Ten studies (58.8%) reported the antimicrobial susceptibility method was applied: disc diffusion method only [18, 21, 28–30, 32, 33], or disc diffusion method and Etest (Epsilometer test) [25, 26, 31]. Five of them described guidelines they used to check laboratory quality (3 in accordance to the Clinical Laboratory Standards Institute [25, 26, 32] and 2 in accordance to the external quality assurance program of the United Kingdom National External Quality Assessment Service [23, 33]).

### Demographics

**Fig. 2:**
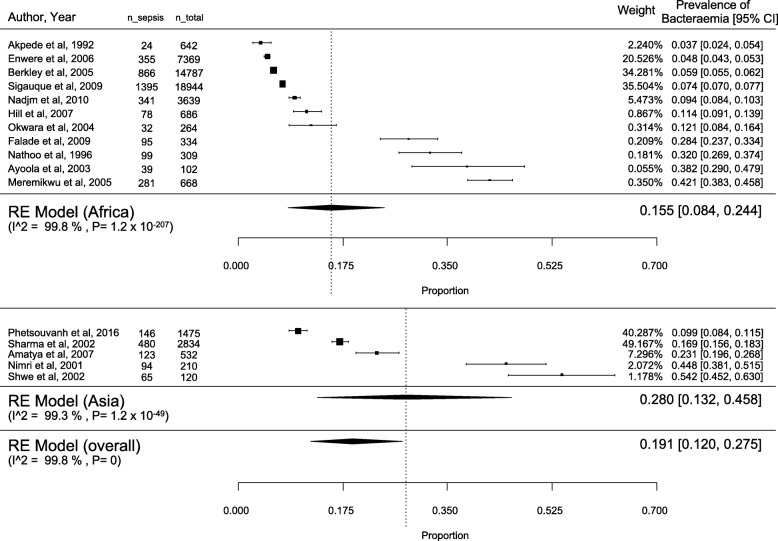
Proportion of bacteraemia in the included studies

A total of 61,015 children were included in the initial review. Of these, 9818 children were from Asian countries (16.1%) and 51,197 from African countries (83.9%). During the data extraction, we excluded 8100 patients as their ages were less than 1 month or more than 18 years. Finally, 52,915 children aged between 1 month and 18 years were included in the systematic review. Among these children, 44,859 (84.8%) were from rural district hospitals, and 8056 (15.2%) from urban hospitals and referral centres. The overall rate of positive blood culture was 19.1% [95% confidence interval (CI): 12.0–27.5; I2 = 99.8%], with 15.5% [95% CI: 8.4–24.4; I2 = 99.8%] in Africa and 28.0% [95%CI: 13.2–45.8; I2 = 99.3%] in Asia (Fig. 2). The majority of included studies did not report any data on comorbidities.

Nutrition status was reported in only four studies [18, 22, 25, 33]. Severe malnutrition was significantly associated with bacteraemia in two of them with an odds ratio of 2.02 (95% CI 1.65 to 2.47) [22] and 1.82 (95% 1.60 to 2.08) [21]. In contrast, no association was found between bacteraemia and malnutrition in the 2 other studies [18, 33].

Only 4 studies [18, 22, 26, 33] included HIV antibody testing and reported the results, of which two studies described an association between HIV infection and bacteraemia with an odds ratio of 3.22 (95% CI: 2.34–4.44) [22] and 2.68(95% CI: 1.55 to 4.64) [18].

Similarly, the prevalence of concurrent malaria was poorly described: four studies [17, 20, 25, 33] reported prevalence of malaria parasitaemia with species unspecified.

**Fig. 3:**
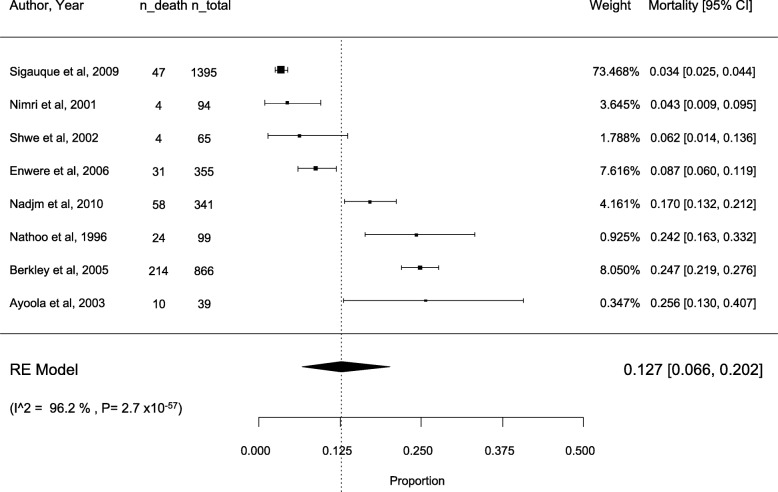
All-cause mortality rate in the included studies

Eight studies [18, 19, 22, 23, 25–28] reported the all-cause fatality rate amongst patients with a bloodstream infection. The overall estimate for all-case fatality rate was 12.7% [95%CI: 6.6–20.2; I2 = 96.2%] (Fig. 3).

### Sources of infection

Only 3 studies reported the suspected underlying source of infection [24, 27, 33]. Of these studies, pneumonia was reported as the first source of infection in 2 studies (32 and 43%) [24, 33]. In 1 study [27], the focal source of bacteraemia were gastroenteritis (40.4%), pneumonia (20%), meningitis (7.4%) and urinary tract infections (7.4%).

### Bacterial identification

**Fig. 4:**
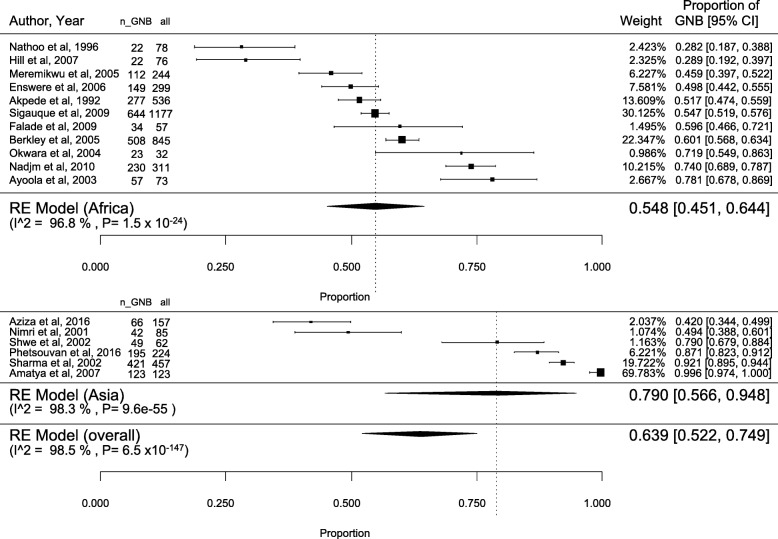
Proportion of Gram-negative bacteria (GNB) in paediatric bacteraemia

A total of 4836 bacterial isolates were included in the studies. Of these, 2974 were Gram-negative (63.9% [52.2–74.9]; I2 = 98.5%) (Fig. 4) and 1858 were Gram-positive (35.8% [24.9–47.5]; I2 = 98.4%) (Additional file 2: Figure S1). Four pathogens were classified as other species. The most commonly isolated pathogens were *S. aureus, S. pneumoniae, E. coli*, non-typhoidal *Salmonella* and *S.* typhi (Table 1).

**Table 1:** Meta-analysis estimating the average proportion (in %) of each pathogen involved in the studies included and by continent

Pathogens	Overall (*n* = 17)				Africa (*n* = 11)				Asia (*n* = 6)			
	Proportion (RE model)	95% CI	I^2^ (%)	*p* value	Proportion (RE model)	95% CI	I^2^ (%)	*p* value	Proportion (RE model)	95% CI	I^2^ (%)	*p* value
*E. coli*	8.8	5.3–12.9	94.7	1.8E-55	10.7	5.8–16.7	95.9	1.6E-43	5.5	2.4–9.7	83.1	0.001
non typhoidal *Salmonella*	6.5	2.1–12.8	98.1	2.0E-214	9.8	3.1–19.4	98.4	3.0E-138	2.1	0.0–8.1	94.9	9.7E-21
*S.* Typhi	6.1	0.3–17.5	99.3	8.4E-278	0.5	0.0–1.2	73.4	0.0002	26.2	0.8–68.5	99.5	1.1E-206
*Klebsiella* sp*.*	3.3	1.0–6.9	96.3	8.5E-100	1.8	0.2–4.6	94.5	3.2E-11	6.6	0.8–16.6	96.1	2.0E-44
Unspecified *Salmonella*	2.6	0.2–6.7	97.7	1.8E-124	2.3	0.0–7.6	97.9	9.1E-22	2.9	0.0–12.3	97.0	3.1E-67
*H. influenzae*	2.3	0.8–4.6	93.4	6.0E-71	2.8	0.6–6.2	95.1	6.5E-55	1.5	0.0–4.4	85.0	6.7E-06
*Pseudomonas sp.*	1.8	0.8–3.2	85.0	1.9E-17	0.90	0.3–1.8	70.8	3.5E-05	3.9	1.4–7.5	82.8	6.1E-06
*Acinetobacter sp.*	1.0	0.4–2.0	79.3	2.2E-14	1.0	0.2–2.2	82.2	7.9E-12	1.1	0.1–3.0	72.7	0.0001
*Enterobacter sp.*	0.5	0.3–1.0	51.8	0.007	0.5	0.1–1.1	62.7	0.001	0.7	0.2–1.5	16.6	0.54
*N. meningitidis*	0.3	0.0–0.6	47.8	0.007	0.2	0.0–0.6	46.3	0.032	0.6	0.0–2.0	66.6	0.02
other GNB	8.9	4.1–15.3	97.6	4.3E-114	10.5	4.2–19.0	97.9	6.6E-80	6.4	0.5–17.5	96.7	8.2E-38
all GNB	63.9	52.2–74.9	98.5	6.5E-147	54.8	45.1–64.4	96.8	1.5E-24	79.0	56.7–94.8	98.3	9.7E-55
*S. aureus*	13.8	7.7–21.2	97.7	7.3E-143	17.8	8.7–29.2	98.3	9.3E-134	7.7	3.3–13.6	89.0	3.2E-07
*S. pneumoniae*	12.6	6.3–20.7	98.1	1.8E-175	16.8	8.5–27.3	98.1	2.2E-84	6.3	0.3–17.9	97.0	2.3E-30
other *Streptococcus*	1.2	0.3–2.4	87.1	1.7E-18	1.0	0.2–2.2	82.7	4.4E-11	1.6	0.0–5.2	89.6	8.9E-10
Group A S*treptococcus*	0.7	0.2–1.4	71.5	1.2E-08	0.7	0.1–1.6	74.5	2.7E-07	0.7	0.0–2.1	66.1	0.01
*Enterococcus sp.*	0.3	0.0–0.8	66.9	0.0004	0.01	0.0–0.2	20.2	0.015	0.7	0.0–2.4	74.3	0.006
other GPB	1.4	0.4–2.9	89.6	9.2E-21	1.6	0.2–4.0	93.2	1.1E-19	1.13	0.1–3.0	72.2	0.002
all GPB	35.8	24.9–47.5	98.4	3.8E-146	45.18	35.6–55.0	96.8	1.5E-24	20.4	5.2–41.9	98.2	2.7E-52
other species	0.1	0–0.3	26.0	0.097	0	0.0–0.06	0.0	0.46	0.48	0.0–1.5	54.0	0.06

Abbreviations: *RE* random effect; 95%CI, 95% confidence interval; *GNB* Gram negative bacteria, *GPB* Gram positive bacteria

Gram-negative bacteria predominance was higher in Asia (79.0% [56.7–94.8]; I2 = 98.3%) than in Africa (54.8% [45.1–64.4]; I2 = 96.8%]. In Asia, *Salmonella* typhi (26.2% [0.75–68.47]; I2 = 99.5%) ranked first followed by *S. aureus* (7.7% [3.3–13.6]; I2 = 89%), *Klebsiella* sp*.* (6.6% [0.8–16.6]; I2 = 96.1%), *S. pneumoniae* (6.3% [0.3–17.9]; I2 = 97.1%), and *E. coli* (5.5% [2.4–9.7]; I2 = 83.1%), whereas in Africa, *S. aureus* (17.8% [8.7–29.2]; I2 = 98.3%) and *S. pneumoniae* (16.8% [8.5–27.3]; I2 = 98.1%) were predominant followed by *E. coli* (10.7% [5.8–16.1]; I2 = 95.9%) and non-typhoidal *Salmonella* (9.8% [3.1–19.4]; I2 = 98.4%) (Table 1). No significant trends on the variation of the proportion of the main pathogens in both continents were observed by year of publication (data not shown).

Pathogens thought to be contaminants were explicitly reported to have been excluded from the analyses in 8 studies [18, 20, 22, 23, 25, 26, 32, 33]. In 6 studies [17, 21, 27, 29–31] providing full data, contaminants were isolated from 132 (2%) blood cultures. These included 111 *Staphylococcus epidermidis*, 16 *Streptococcus viridans*, and 4 *Bacillus cereus*.

### Antibiotic susceptibility testing

We obtained antimicrobial susceptibility data from 6 studies in Africa [18, 21, 23, 25, 26, 33] and 5 studies from Asia [28–32] that reported detailed antimicrobial susceptibility test (AST) results, including a total of 3078 isolates (2194 isolates in Africa and 884 isolates in Asia).

Overall, we observed antimicrobial resistance rates of 59.7% (1988/3328) to ampicillin, 33.5% (769/2295) to gentamicin, 34.9% (287/822) to amikacin, 45.3% (1518/3348) to chloramphenicol, 49.0% (1371/2800) to cotrimoxazole, 33.9% (496/1464) to third-generation cephalosporins, and 43.3% (385/890) to ciprofloxacin. Table 2 shows the number of isolates of the major pathogens that were susceptible to the main antibiotics. We observed that *S. aureus* was more likely to be resistant to methicillin (oxacillin, third-generation cephalosporins) in Africa than in Asia (29.5% vs. 7.9%, respectively) as well as to cotrimoxazole (49.7% vs. 20.3%, respectively). *S. pneumoniae* exhibited high susceptibility to beta-lactams in Africa, whereas 22% of isolates were resistant to ampicillin in Asia (Table 2). *E. coli* and *Klebsiella* sp. were more frequently resistant to the 3rd generation cephalosporins, aminoglycosides, and ciprofloxacin in Asia than in Africa (Table 2). In Asia, about one third of *S.* typhi isolates were resistant to first-line antibiotics (33.3% to ampicillin, 32.7% to chloramphenicol and 16% to cotrimoxazole), 7% to third-generation cephalosporins, and 14% to ciprofloxacin (26% revealed resistance to nalidixic acid) (Table 2). It was not possible to identify trends in resistance rates over time, due to the lack of standard reporting between studies.

**Table 2:** Antibiotic susceptibility tests by pathogen and continents

		Ampicillin	Third generation cephalosporins	Gentamicin	Amikacin	Chloramphenicol	Cotrimoxazole	Ciprofloxacin	Nalidixic acid	Clindamycin
*S. aureus*	Africa	37/369 (10.0)	91/129 ^a^ (70.5)	372/414 (89.9)	0/0 (−)	234/373 (62.7)	180/351 (51.3)	0/0 (−)	0/0 (−)	16/22 (72.7)
	Asia	12/77 (15.6)	39/43 ^a^ (90.7)	155/186 (83.3)	63/81 (77.8)	66/85 (77.6)	63/79 (79.7)	0/0 (0.0)	0/0 (−)	55/76 (72.4)
	Total	49/446 (11.0)	130/172 ^a^ (75.6)	527/600 (87.8)	63/81 (77.8)	300/458 (65.5)	243/430 (56.5)	0/0 (−)	0/0 (−)	71/98 (72.4)
*S. pneumoniae*	Africa	531/569 ^b^ (93.3)	132/132 (100.0)	2/20 (10.0)	0/0 (−)	556/605 (91.9)	347/596 (58.2)	0/0 (−)	0/0 (−)	20/20 (100.0)
	Asia	25/32 ^b^ (78.1)	0/0 (−)	0/0 (−)	0/0 (−)	4/4 (100.0)	1/5 (20.0)	0/0 (−)	0/0 (−)	0/0 (−)
	Total	556/601 ^b^ (92.5)	132/132 (100.0)	2/20 (10.0)	0/0 (−)	560/609 (92.0)	348/601 (57.9)	0/0 (−)	0/0 (−)	20/20 (100.0)
*E. coli*	Africa	13/236 (5.5)	52/66 (78.8)	183/265 (69.1)	8/8 (100.0)	79/281 (28.1)	37/251 (14.7)	9/9 (100.0)	0/0 (−)	–
	Asia	27/105 (25.7)	64/93 (68.8)	37/66 (56.1)	38/54 (70.4)	41/109 (37.6)	20/55 (36.4)	38/60 (63.3)	46/53 (86.8)	–
	Total	40/341 (11.7)	116/159 (73.0)	220/331 (66.5)	46/62 (74.2)	120/390 (30.8)	57/306 (18.6)	47/69 (68.1)	46/53 (86.8)	–
*Klebsiella* sp*.*	Africa	0/20 (0.0)	0/0 (−)	16/19 (84.2)	8/8 (100.0)	7/19 (36.8)	6/18 (33.3)	0/0 (−)	0/0 (−)	–
	Asia	30/489 (6.1)	123/477 (25.8)	93/480 (19.4)	247/471 (52.4)	100/489 (20.4)	12/18 (66.7)	183/471 (38.9)	15/18 (83.3)	–
	Total	30/509 (5.9)	123/477 (25.8)	109/499 (21.8)	255/479 (53.2)	107/508 (21.1)	18/36 (50.0)	183/471 (38.9)	15/18 (83.3)	–
non-typhoidal *Salmonella*	Africa	196/654 (30.0)	94/94 (100.0)	488/564 (86.5)	9/10 (90.0)	316/656 (48.2)	232/646 (35.9)	7/7 (100.0)	0/0 (−)	–
	Asia	36/44 (81.8)	39/39 (100.0)	0/0 (−)	0/0 (−)	6/7 (85.7)	39/44 (88.6)	35/36 (97.2)	7/7 (100.0)	–
	Total	232/698 (33.2)	133/133 (100.0)	488/564 (86.5)	9/10 (90.0)	322/663 (48.6)	271/690 (39.3)	42/43 (97.7)	7/7 (100.0)	–
*S.* Typhi	Africa	–	–	–	–	–	–	–	–	–
	Asia	333/499 (66.7)	300/323 (92.9)	139/190 (73.2)	162/190 (85.3)	332/493 (67.3)	279/332 (84.0)	210/245 (85.7)	202/274 (73.7)	–
	Total	333/499 (66.73)	300/323 (92.9)	139/190 (73.2)	162/190 (85.3)	332/493 (67.3)	279/332 (84.0)	210/245 (85.7)	202/274 (73.7)	–
*H. influenzae*	Africa	80/157 (50.95)	0/0 (−)	22/23 (95.7)	0/0 (−)	70/147 (47.6)	204/388 (52.6)	0/0 (−)	0/0 (−)	–
	Asia		–	–	–	–	–	–	–	–
	Total	80/157 (50.95)	0/0 (−)	22/23 (95.7)	0/0 (−)	70/147 (47.6)	204/388 (52.6)	0/0 (−)	0/0 (−)	–

Results are shown as: n susceptible isolates/n isolates tested (%) ^a^In 2 studies [32, 33], we made the assumption that oxacillin susceptible isolates were susceptible to 3rd geneneration cephalosporins, whereas susceptibility to 3rd geneneration cephalosporins was specifically tested in 2 other studies [21, 28]

^b^ *S. pneumoniae* isolates have been tested to penicillin

## Discussion

To our knowledge, this is the largest systematic review that has characterized the pathogen distribution and antimicrobial resistance patterns in paediatric bacteraemia in LMICs including 52,915 children and 4836 isolates. We identified a high rate of positive blood cultures (19.1%), while previous studies have observed a percentage of positive blood culture from 7 to 13.9% [7, 34]. Gram-negative bacteria accounted for 63.9% of all episodes, and *Salmonella spp.* was the most common pathogen reported in Asia (31.1%). *S. aureus* and *S. pneumoniae* were more predominant in Africa.

There was a high overall antimicrobial resistance rate to the first-lines drugs (ampicillin and gentamicin) but also to the second line recommended therapy (3rd generation cephalosporins and amikacin).

Of interest, we observed a marked variation of resistance patterns between Asia and Africa. Indeed, *S. aureus* was more commonly resistant to methicillin in Africa, whereas *E. coli* was more frequently resistant to 3rd generation cephalosporins, amikacin and ciprofloxacin in Asia.

Overall, these results are consistent with a previous systematic review in which Gram-negative bacteria accounted for 66.8% of sepsis cases in resource-limited countries, with *Salmonella* spp. as predominant cause of sepsis in Asia [7]; whereas Gram-positive pathogens were more likely involved in high-income countries [35]. The high levels of resistance we observed are similar to those previously described in Sub-Saharan Africa and Asia. Indeed, in a recent systematic review [10], prevalence of bacteria susceptible to the penicillin and gentamicin combination, to chloramphenicol, and to third generation cephalosporins was 63, 47 and 64%, respectively among older infants with bacteraemia.

The overall all-cause mortality rate in this review was 12.7% [6.6–20.2]. Although difference in mortality due to sepsis is most likely related to the higher prevalence of comorbidities such as malaria, malnutrition, or immunosuppression [36], we found very few studies that included HIV-infected children [18, 22, 33] despite the fact that HIV infection is the most common cause of morbidity and mortality in children in sub-Saharan Africa [18]. Data on all the comorbidities associated with bacteraemia are scarce in the studies; it is difficult to draw conclusions.

Moreover, no information was available about other factors that may contribute to risk of mortality from bacteraemia, including access to health care, vaccination status, late clinical diagnosis, concordant versus discordant empiric treatment, time to first dose of antibiotics and time to switch from empiric to targeted therapy. Indeed, for optimal outcomes, treatment of sepsis is time sensitive and should be started before laboratory data confirm the diagnosis [37]. Moreover, in a study from Thailand conducted in 2010, it was estimated that around an extra 19,000 deaths were caused by multi-drug resistant bacteria each year [38] . The mortality attributable to ESBL-producing pathogens and methicillin-resistant *S. aureus* (MRSA) is estimated to be 27 and 34% in neonatal sepsis in Tanzania, respectively [39], which has been used to estimate that 58,319 deaths could be attributable to ESBL and MRSA in India alone [40].

### Strength and limitations

To the best of our knowledge, this is the largest systematic review to date describing the pathogen distribution and AMR patterns amongst children with bacteraemia across LMIC settings. However, there are several limitations to be addressed. First, this review includes only 17 studies combining data from 4706 episodes of bacteraemia from 12 countries, representing 9% (12/137) of the LMIC defined in The World Bank classification [12]. The estimated rates of bacteraemia and all-cause mortality as well as pathogen distribution and AMR patterns are therefore not representative of the entire region.

Secondly, we observed high heterogeneity between studies that may be ascribed to the heterogeneity of the study designs but also the timeframe as studies spanned a period of more than 20 years, so inclusion criteria, hospital type, population characteristics varied considerably, which raises concerns about the appropriateness of our aggregation of the different datasets. However, we attempted to control heterogeneity by specifying predefined inclusion criteria and by using a random-effect meta-analysis model. We further performed subgroup analyses by continents (Asia and Africa) to explore the effect of heterogeneity on the overall prevalence of bloodstream infections and for each pathogen. Unfortunately, we did not observe a decrease of the heterogeneity by subgroup analyses. Concerningly only limited recent data could be identified, since the most recent study we included was published in 2016 on data collected between 2001 and 2011 [32]. Our strict inclusion criteria may have led to exclusion of papers in which the distribution of pathogens could not be distinguished from neonates to older children. Similarly, South American studies were either missed by the search strategy or did not contain any data met the study inclusion criteria. In addition, as we did not use concrete names of countries, some other studies from LMIC that did not use LMIC or similar terms may have been missed by the search strategy.

Thirdly, the search strategy was made with two major scientific databases and included only English-language literature. These two points may have led to selection bias since the articles of interest may not have been published in English or in these two databases.

Fourthly, the quality assurance practices of clinical microbiology labs, particularly in rural lab settings of low income countries, are likely not of equal proficiency and the identification and particularly the susceptibility testing results may be different between studies. For example, reporting aminoglycosides susceptibility for *Salmonella* species in 6 studies [18, 25, 26, 28, 29, 33] is concerning (these agents are not considered clinically useful against *Salmonella* species). Although WHO [41] recommends laboratory results as accurate as possible, with all aspects of the laboratory operations must be reliable, in our study, only five studies described guidelines they used to check laboratory quality [23, 25, 26, 32, 33].

Then, denominators for every organism differ across the tested antibiotics (i.e., 446 *S. aureus* were tested to ampicillin, 600 to gentamicin and only 172 to cephalosporins), that may suggest heterogeneity between laboratory practices. However, given the extremely limited data available, we chose to rather include these studies in our review, but to acknowledge the above mentioned limitations. Quality in reporting practices are also important in order to be useful in a clinical or public health setting. The challenge is to reduce the level of inaccuracy as much as possible, given the limitations of our testing systems.

Fifthly, our review revealed a very high resistance of *Klebsiella* sp*.* in Asia, which should be interpreted with caution because 96% of *Klebsiella* sp. bacteraemia episodes included in this review were retrieved from one single study based in India [29]. A previous study reported that India had the highest rates of paediatric ESBL rates in Asia [42]. A retrospective analysis of neonatal Gram-negative septicemia from 2002 to 2003 reported that 61% (46/75) of cases were due to ESBL-producing strains [43].

## Conclusions

As defined by the World Health Assembly in 2017 [44], improving the understanding of the epidemiological and financial burden of sepsis worldwide is a priority. In our review, we focused on description of paediatric bacteraemia and and we identified a major lack of recent high quality data from LMIC settings. There is a clear need for new prospective studies of paediatric community-acquired sepsis that utilize standardized definitions. These future studies should describe the clinical presentation, identify the infection source, document underlying co-morbidities and factors associated with mortality of children presenting with bacteremia.

Similarly, recent data on the antimicrobial resistance patterns are essential to accurately evaluate the appropriateness of currently-recommended empiric and targeted antibiotic therapies. Our results suggest that new empiric treatment regimens and strategies for enhanced prevention of paediatric bacteraemia and other serious bacterial infections are needed to reduce attributable child mortality.

## Supplementary information

**Additional file 1: **Search strategy used in Embase. **Table S1.** a Summary of studies on bloodstream infections in Africa included in the review, 1990–2017. b Summary of studies on bloodstream infections in Asia included in the review, 1990–2017.

**Additional file 2: Figure S1.** Proportion of Gram positive bacteria (GPB) in paediatric sepsis.

## Data Availability

This review was based on data extracted from published papers available in the public domain.

## Abbreviations

AST: Antimicrobial susceptibility test
ESBL: Extended-spectrum beta-lactamase
Etest: Epsilometer test
GNB: Gram-negative bacteria
LMIC: Low- and middle-income countries
MeSH: Medical Subject Headings
MRSA: Methicillin-resistant *S. aureus*
STROBE: STrengthening the Reporting of OBservational studies in Epidemiology
WHO: World Health Organization
