# Supplementary material for: Bacterial pathogens and resistance causing community acquired paediatric bloodstream infections in low- and middle-income countries: a systematic review and meta-analysis
Source: Antimicrob Resist Infect Control. 2019 Dec 30;8:207. doi: 10.1186/s13756-019-0673-5 (PMC6937962; doi:10.1186/s13756-019-0673-5)
Supplement: Supplementary file 1 — Additional file 1: Search strategy used in Embase. Table S1. a Summary of studies on bloodstream infections in Africa included in the review, 1990–2017. b Summary of studies on bloodstream infections in Asia included in the review, 1990–2017. [file 13756_2019_673_MOESM1_ESM.docx]

**Additional file 1**

**Search strategy used in Embase**

Database: Embase <1974 to 2019 Week 46>

Search Strategy:

--------------------------------------------------------------------------------

1 bacteremia/ (39937)

2 septicemia/ (18299)

3 septicemia/ (18299)

4 limit 3 to (human and english language and yr="2018 - 2019") (1094)

5 sepsis/ (149317)

6 limit 5 to (human and english language and yr="2018 - 2019") (16198)

7 fever/ (224594)

8 limit 7 to (human and english language and yr="2018 - 2019") (27297)

9 bloodstream infection/ (12064)

10 limit 9 to (human and english language and yr="2018 - 2019") (2418)

11 1 or 4 or 6 or 8 or 10 (83396)

12 developing country/ (92071)

13 limit 12 to (human and english language and yr="2018 - 2019") (3848)

14 under-developed nations.mp. (7)

15 limit 14 to (human and english language and yr="2018 - 2019") (0)

16 third world nation.mp. (6)

17 limit 16 to (human and english language and yr="2018 - 2019") (0)

18 Resource limited setting.mp. (1630)

19 limit 18 to (human and english language and yr="2018 - 2019") (366)

20 low middle income countr*.mp. (1230)

21 limit 20 to (human and english language and yr="2018 - 2019") (441)

22 13 or 15 or 17 or 19 or 21 (4596)

23 11 and 22 (186)

24 limit 23 to (english language and embase and yr="2018 - 2019" and (infant <to one year> or child <unspecified age> or preschool child <1 to 6 years> or school child <7 to 12 years> or adolescent <13 to 17 years>)) (29)

**Table S1a**: Summary of studies on bloodstream infections in Africa included in the review, 1990-2017

| **Author, year** | **Location** | **Study period** | **Hospital type** | **Inclusion criteria** | **Blood volume taken for bacterial culture** | **Age group** | **Community acquired pathogens only** | **Patients who had a blood sample taken (n) *** | **Patients with a positive blood culture (n; %) *** | **Case fatality (%)** | **Most common identified isolates** | **Quality Assessment (ratio of STROBE items)** |
| --- | --- | --- | --- | --- | --- | --- | --- | --- | --- | --- | --- | --- |
| **Akpede et *al*, 1992** | Nigeria, Benin city | Oct 1988 to Oct 1989 | Urban teaching hospital | Fever without localising signs | _ | 1 mo to 5 yrs | Yes | 642 | 24 (4) | _ | *E. coli, S. aureus* | 66.7 |
| **Nathoo *et al*, 1996** | Zimbabwe, Harare | Jul 1993 to Dec1994 | Urban referral hospital | Fever | 1-3ml | 1 mo to 8 yrs | Yes | 309 | 99 (32) | 24/99 (24) | *S. aureus, S. pneumoniae* | 69.7 |
| **Ayoola *et al*, 2003** | Nigeria, Ibadan | Jun 1998 to Nov 1998 | Urban referral and teaching hospital | Fever | 2 ml | 1 mo to 12 mo | Yes | 102 | 39 (38) | 10/39 (26) | *E. coli, S. aureus* | 72.7 |
| **Okwara *et al,* 2004** | Kenya, Nairobi | Janu 2001 to Mar 2001 | Urban teaching hospital | Fever without localising signs | 2 ml | 3 mo to 12 yrs | Yes | 264 | 32 (12) | _ | *Salmonella spp* | 69.7 |
| **Meremikwu *et al,* 2005** | Nigeria, Calabar | Jul 1996 to Dec 2002 | Tertiary hospital, teaching hospital | Fever without localising signs | _ | 2 mo to 18 yrs | Yes | 668 | 281 (42) | _ | *E. coli, S. aureus* | 51.5 |
| **Berkley *et al*, 2005** | Kenya, Kilifi | Aug 1998 to Jul 2002 | Rural hospital | All hospital or ward admissions, irrespective of fever | 1-2 ml | 2 mo to 13 yrs | Yes | 14787 | 866 (6) | 214 /866 (25) | *non-*typhoidal *Salmonella, H. Influenzae, S. pneumoniae* | 79.4 |
| **Enwere *et al*, 2006** | Gambia, Basse and Bansang. | Aug 2000 to Apr 2004 | Rural Hospital | Fever | _ | 2 mon to 29 mo | Yes | 7369 | 355 (5) | 31/355 (9) | *non-*typhoidal *Salmonella, S. pneumoniae* | 66.7 |
| **Hill e*t al*, 2007** | Gambia, Banjul | Nov 2003 to Feb 2005 | Urban hospital | Fever without localising signs | _ | 2 mo to 15 yrs | Yes | 686 | 78 (11) | _ | *S. pneumoniae, S. aureus* | 75.8 |
| **Falade *et al*, 2009** | Nigeria, Ibadan | Feb 2005 to Jun 2007 | Urban teaching hospital | Suspect pneumococcal disease (pneumonia, meningitis, and septicemia) | _ | 2 mo to 59 mo | Yes | 334 | 95 (28) | _ | *Salmonella spp, S. aureus* | 69.7 |
| **Sigauque *et al* *,*2009** | Mozambique, Manhiça | May 2001 to Apr 2006 | Rural Hospital | All admissions, except trauma | 1-3ml | 1 mo to 14 yrs | Yes | 18944 | 1395 (7) | 147 /1395 (10) | *non-*typhoidal *Salmonella, .S pneumoniae* | 53.1 |
| **Nadjm et *al,* 2010** | Tanzania, Tanga | Jun 2006 to May 2007 | Rural hospital | Fever | 2-5ml | 2 mon to 13 yrs | Yes | 3639 | 341 (9) | 58/341 (17) | *non-*typhoidal *Salmonella, S. pneumoniae* | 81.3 |

*, After exclusion of neonate population, depending on age grouping of the studies

**Table S1b**: Summary of studies on bloodstream infections in Asia included in the review, 1990-2017

| **Author, year** | **Location** | **Study period** | **Hospital type** | **Inclusion criteria** | **Blood volume taken for bacterial culture** | **Age group** | **Community acquired pathogens only** | **Patients who had a blood sample taken (n) *** | **Patients with a positive blood culture (n; %) *** | **Case fatality (%)** | **Most common identified isolates** | **Quality Assessment (ratio of STROBE items)** |
| --- | --- | --- | --- | --- | --- | --- | --- | --- | --- | --- | --- | --- |
| **Nimri *et al*, 2001** | Jordan, Irbid | Jan 1998 to Dec 1999 | Paediatric referal hospital | Fever | _ | 1 mo to 10 yrs | 10% were suspected to be nosocomial | 210 | 94 (45) | 4/94 (4) | *S. pneumoniae* | 56.3 |
| **Shwe *et al*,2002** | Myanmar, Yangon | Aug1998 to Jul 1999 | One's children, rural hospital | Fever>5 days, non-antibiotic use past 48h | 5 ml | 1mo to 12 yrs | Yes | 120 | 65 (54) | 4/65 (6) | *S.* Typhi | 56.7 |
| **Sharma *et al*, 2002** | India, Rohtak | Jan 2001 to Jan 2002 | Tertiary hospital | Fever or other clinical features of sepsis | 2-3ml | 1mo to 14 yrs | Yes | 2834 | 480 (17) | _ | *Salmonella spp , Klebsiella sp* | 50.0 |
| **Amatya *et al*, 2007** | Nepal, Kathmandu | Apr 2005 to Jun 2005 | Tertiary referral hospital | Febrile, suspected bacteraemia | 3 ml | 2 mo to 15 yrs | Yes | 532 | 123 (23) | _ | *S.* Typhi | 56.7 |
| **Phetsouvanh *et al*, 2016** | Laos, Ventiane | Feb 2000 to Feb 2004 | Tertiary hospital | Admitted with suspected community acquired bacteraemia | 1-2ml | 1 yr to 15 yrs | Yes | 1475 | 146 (10) | _ | *S.* Typhi*, S. aureus* | 62.5 |
| **Nor Azizah *et al*, 2016** | Malaysia, Selangor | Jan 2001 to Dec 2011 | Tertiary government hospital | Admitted with suspected community acquired bacteraemia | _ | 1 mo to 13 yrs | Yes | _ | 193 | _ | non-typhoidal *Salmonella*, *S. pneumoniae, S. aureus* | 78.1 |

*, After exclusion of neonate population, depending on age grouping of the studies
