# Supplementary figures and images for: Bacterial pathogens and resistance causing community acquired paediatric bloodstream infections in low- and middle-income countries: a systematic review and meta-analysis
Source: Antimicrob Resist Infect Control. 2019 Dec 30;8:207. doi: 10.1186/s13756-019-0673-5 (PMC6937962; doi:10.1186/s13756-019-0673-5)

# Supplemental Figure. Proportion of Gram positive bacteria (GPB) in paediatric sepsis

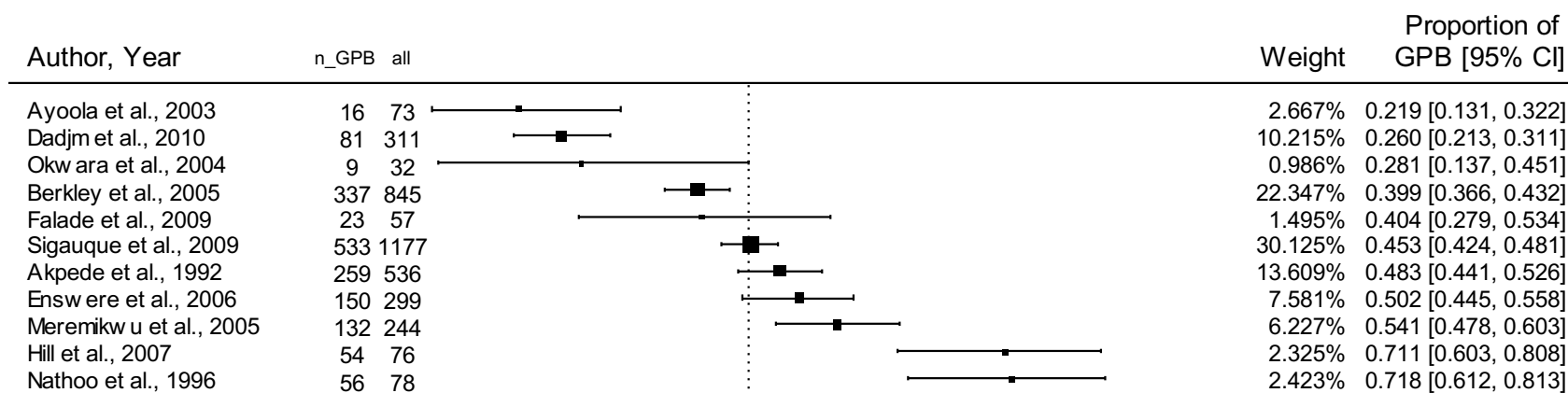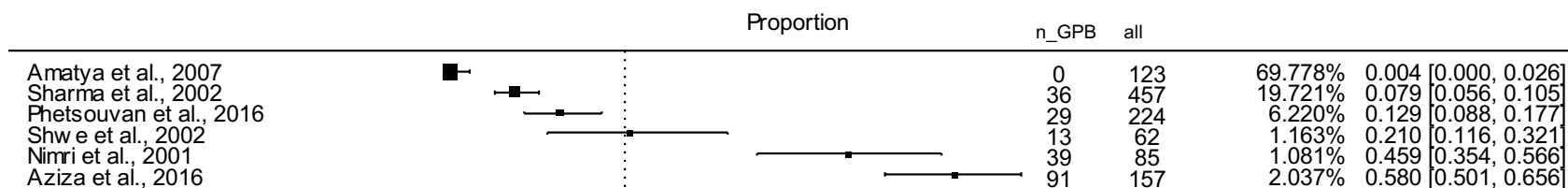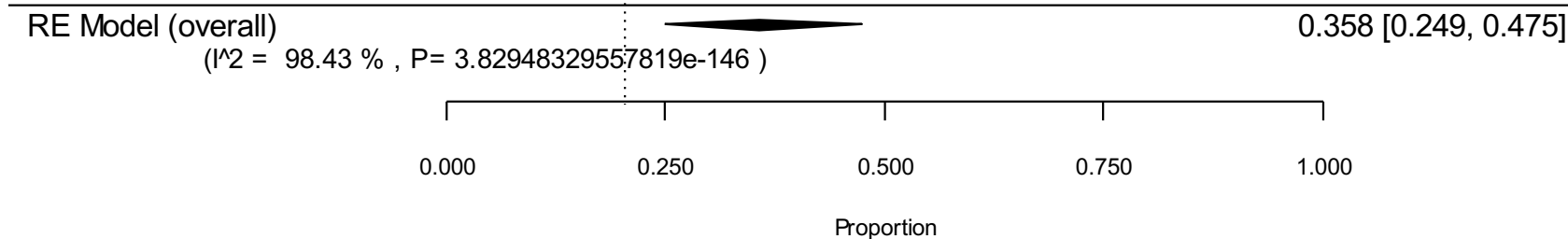

Supplement: Supplementary file 2 — Additional file 2: Figure S1. Proportion of Gram positive bacteria (GPB) in paediatric sepsis. [file 13756_2019_673_MOESM2_ESM.pdf]
